# Targeting NLRP3 Inflammasome in the Treatment Of Diabetes and Diabetic Complications: Role of Natural Compounds from Herbal Medicine

**DOI:** 10.14336/AD.2021.0318

**Published:** 2021-10-01

**Authors:** Ying Bai, Qianqian Mu, Xueli Bao, Jiacheng Zuo, Xin Fang, Jing Hua, Dongwei Zhang, Guangjian Jiang, Ping Li, Sihua Gao, Dandan Zhao

**Affiliations:** ^1^College of Traditional Chinese Medicine, Beijing University of Chinese Medicine, Beijing, China; ^2^Dongzhimen Hospital, Beijing University of Chinese Medicine, Beijing, China; ^3^Third Affiliated Hospital, Beijing University of Chinese Medicine, Beijing, China

**Keywords:** NLRP3 inflammasome, diabetes, diabetic complications, natural compounds, herbal medicine

## Abstract

Diabetes, a common metabolic disease with various complications, is becoming a serious global health pandemic. So far there are many approaches in the management of diabetes; however, it still remains irreversible due to its complicated pathogenesis. Recent studies have revealed that nucleotide-binding and oligomerization domain-like receptor family pyrin domain-containing 3 (NLRP3) inflammasome plays a vital role in the progression of diabetes and many of its complications, making it a promising therapeutic target in pharmaceutical design. Natural derived herbal medicine, known for its utilization of natural products such as herbs or its bioactive ingredients, is shown to be able to ameliorate hyperglycemia-associated symptoms and to postpone the progression of diabetic complications due to its anti-inflammatory and anti-oxidative properties. In this review, we summarized the role of NLRP3 inflammasome in diabetes and several diabetic complications, as well as 31 active compounds that exert therapeutic effect on diabetic complications via inhibiting NLRP3 inflammasome. Improving our understanding of these promising candidates from natural compounds in herbal medicine targeting NLRP3 inflammasome inspires us the relationship between inflammation and metabolic disorders, and also sheds light on searching potential agents or therapies in the treatment of diabetes and diabetic complications.

Diabetes is characterized by increased blood glucose due to insufficient or lack of insulin production, or inability of peripheral tissues to respond to insulin (insulin resistance, IR). Latest data from International Diabetes Federation (IDF) reveals that there are currently 463 million adults living with diabetes, and approximately 4.2 million people died of diabetes and its various complications [1]. The prevalence of diabetes has reached to 9.3% in 2019 and this number is anticipated to keep roaring globally to 10.9% by 2045 [1], making it a severe public health concern that endangers human health and life span. Scientists around the world have been working on exploring therapeutic strategies or potential treatments in the management of diabetes. And so far, we have been witnessing some breakthroughs; yet there is still a long way to go.

Inflammation is a double-edged sword for human health. Moderate inflammatory response is necessary for the body to fight against pathogenic microorganism infection and eliminate toxins within the body, while the dysregulated inflammation leads to tissue and organ damage [2]. In the complicated pathogenic mechanisms of diabetes, chronic low-grade inflammation is considered to be a crucial event. The concept of inflammasome was firstly proposed by Tschopp’s team in 2002 [3]. In recent years, with more and more research about innate immunity and cellular signal transduction being carried out, inflammasomes have become the focus of this field. Among all the inflammasome family members, the nucleotide-binding and oligomerization domain-like receptor family pyrin domain-containing 3 (NLRP3) inflammasome is the most extensively studied and the most characteristic one. It is a tripartite protein and an innate immune sensor of the NOD-like receptors (NLR) family proteins; it can be activated upon stimuli such as infection and intracellular stress and then triggers the maturation of pro-inflammatory cytokines [3-5]. As a molecular switch of inflammatory reaction, NLRP3 is closely related to immune regulation, metabolic disorders, and inflammatory responses to various diseases [6-8]. Clinical and laboratory data both have shown that NLRP3 inflammasome plays a vital role in metaflammation and contributes to the progression of diabetes and several diabetic complications. Thus, NLRP3 inflammasome is proposed to be a potential novel therapeutic target of inflammation related chronic diseases including diabetes [9, 10, 11].[Fig F1-ad-12-7-1587]

**Figure 1. F1-ad-12-7-1587:**
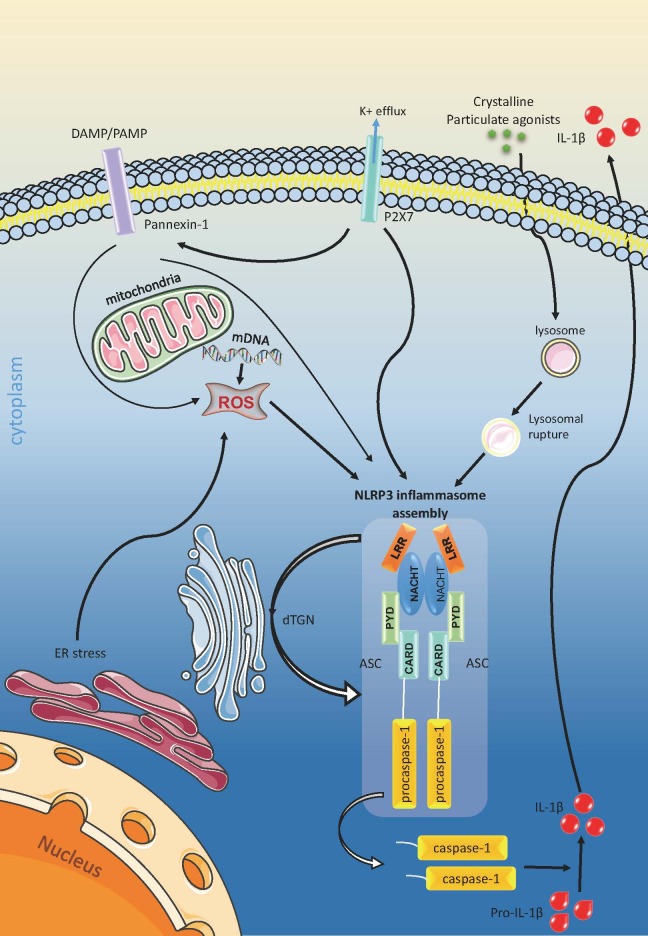
NLRP3 inflammasome activation. (1) K^+^ efflux is sufficient to activate NLRP3. (2) Crystalline or particulate NLRP3 agonists leads to lysosomal rupture after phagocytosis. Then the NLRP3 senses the active enzymes to induce inflammasome assembly in lysosome. (3) Diversified NLRP3 stimuli increases the production of mitochondrial ROS and induces trans-Golgi network (TGN) disassembly into dispersed TGN (dTGN). ROS triggers NLRP3 inflammasome activation, while dTGN acts as scaffold for NLRP3 inflammasome assembly.

Traditional herbal medicine has long been applied and playing a magnificent role in the treatment of diabetes [12, 13]. Meanwhile, active agents from natural medicine are becoming one of the key sources in seeking novel drug candidates, especially those with rich origins, unique structures, and significant activities. Numerous evidence has revealed that herbal medicine exerts anti-diabetic and anti-inflammatory effects in diabetic patients and in rodent models [14, 15]. Lately, quite a few active ingredients of natural herbs and classic prescriptions have been shown to be able to inhibit NLRP3 inflammsome. A previous review has discussed NLRP3 inhibitors [16] however, the authors did not summarize the literature in natural herbal products on NLRP3 inflammsome especially pertinent to diabetes. In this review, we focus on the regulatory mechanisms of NLRP3 inflammasome in diabetes and the recent advances of natural compounds derived from herbal medicine in the management of diabetes and diabetic complications via inhibiting NLRP3 inflammasome.

## NLRP3 inflammasome

### Molecular components and structure of NLRP3 inflammasome

All inflammasomes contain a sensor protein, usually a pattern recognition receptor (PRR), which activates procaspase-1 upon exposure to pathogen associated or risk-related molecular patterns (PAMPs/DAMPs) stimuli [17]. Besides NLRP3, common inflammasomes also include NLRP1, NLRP2, NLRP6, NLRP7, NOD like receiver family card domain containing 4 (NLRC4), absence in melanoma 2 (AIM2), and interferon-γ inducible protein 16 (IFI16) [10]. The NLRs consist 22 members in mammals and they are the core structure of inflammasomes. The latter two inflammasomes are from pyrin and HIN200-hematopoietic interferon-inducible nuclear antigens with 200 amino-acid repeats (PYHIN) family [18].

The canonical NLRP3 inflammasome is made up of three components: the NLRP3 scaffold, the adaptor protein apoptosis-associated speck-like protein containing a caspase-activating and recruitment domain (ASC), and the effector procaspase-1 [19, 20]. Further, the NLRP3 receptor consists three functional domains: The C-terminal leucine-rich repeats (LRRs) that is responsible for identifying and binding PAMPs/DAMPs; the nucleotide-binding and oligomerization domain (NACHT) that is responsible for the formation of oligomers of NLRs; and the N-terminal caspase recruitment (CARD)/pyrin domain (PYD) that mediates downstream signaling transmission [20].

### NLRP3 inflammasome activation

There are mainly three hypotheses about NLRP3 inflammasome activation.

First of all, the ion concentration changes. These include intracellular K^+^ efflux, Ca^2+^ mobilization, as well as Na^+^ influx and Cl^-^ efflux [19, 21]. K^+^ concentration fluctuation was once considered an important event during NLRP3 inflammasome activation, because K^+^ efflux alone is able to activate NLRP3; it also activates Ca^2+^ independent phospholipase A2 that potentiates interleukin -1β (IL-1β) maturation [21, 22]. Notably, recent studies have shown that LPS can activate NLRP3 in mouse macrophages without changing K^+^ concentration, in association with reduced pannexin-1 activity, suggesting that K^+^ efflux is not necessary for activating NLRP3 inflammasome [23]. Cytosolic Ca^2+^, regardless of its source, has been shown to play an important role in NLRP3 activation for its effect on mitochondrial Ca^2+^ overload [24]. Moreover, changes in Na^+^ and Cl^-^ concentration alone does not activate NLRP3 inflammasome, but these ions can modulate the effects of K^+^ efflux [25, 26]. Collectively, decrease in intracellular K? concentration is sufficient but not necessary to activate NLRP3. Changes in Na? and Cl^-^ concentrations and Ca^2+^ mobilization seem to participate in the regulation but are not necessarily sufficient to activate NLRP3 inflammasome by themselves. Secondly, lysosomal rupture. Crystalline or particulate NLRP3 agonists can lead to lysosomal lysis after phagocytosis. Then the NLRP3 senses lysosomal content, especially the active enzymes in the cytoplasm, to induce activation. For example, it has shown that cathepsin-B release is required in NLRP3 inflammasome activation [27-29]. In addition, other cathepsins family proteins (cathepsins C, L, S and Z) have also been implicated in NLRP3 inflammasome activation via ATP or bacterial components, either acting independently or in combination [29].

Last but not least, the mitochondrial reactive oxygen species (mROS). Diversified NLRP3 stimuli can increase the production of mROS, suggesting that mROS might be a common trigger and plays a significant role in the process of NLRP3 inflammasome activation [30-32]. Besides, some researchers have claimed that mitochondria are also closely involved, not only because dysfunctional mitochondria produce ROS, but also that mitochondrial DNA (mDNA) participate in the NLRP3 inflammasome activation [33-35]. However, the specific crosstalk between ROS and NLRP3 inflammasome activation is not fully explored; yet there is evidence that a calcium cation channel transient receptor potential melastatin 2 (TRPM2) might play a linking role in ROS-dependent inflammasome activation [36, 37]. Furthermore, more recent studies have revealed a major mechanism as to how NLRP3 inflammasome corresponds to diversified NLRP3 stimuli. Different NLRP3 agonists all can lead to trans-Golgi network (TGN) disassembly into dispersed TGN (dTGN). Next, NLRP3 is recruited to dTGN by ptdins4p that enriches on the membrane, and finally dTGN serves as a scaffold for NLRP3 inflammasome aggregation and activation. This suggest that dTGN recruitment is possibly the common cellular signal of NLRP3 activation [38].

### NLRP3 inflammasome in diabetes and diabetic complications

Accumulating evidence has shown the intrinsic relationship between NLRP3 inflammasome and diabetes from various aspects. Tschopp et al (2010) first identified NLRP3 inflammasome as a sensor for metabolic danger that might contribute to T2DM progression [4]. This was later supported by the phenotype of greater glucose tolerance and insulin sensitivity in NLRP3^-/-^ mice, which protect the mice from diet-induced obesity and diabetes [31]. These findings showed a potential role of NLRP3 inflammasome in diabetes and diabetic complications; these studies have shed light on diabetes research although a decisive conclusion awaits clarification.

In general, diabetes is a complicated metabolic disorder that requires substantial interaction between genetic susceptibility and environmental factors like exercise and calorie intake. Glucose and saturated fatty acids both are DAMPs that serve as the first signals for upregulation of NLRP3 and pro- IL-1β [39]. Meanwhile, sustained hyperglycemia, increased glycolysis and saturated fatty acids like palmitic acid will also lead to impaired autophagy and mitochondrial dysfunction, which induce mROS overproduction--the second signal activating the NLRP3 inflammasome [19, 40]. Hyperglycemia can also induce overexpression of thioredoxin-interacting protein (TXNIP), which plays a critical role in the TXNIP-dependent NLRP3 inflammasome activation [41]. Besides, gut microbiota caused LPS release may also contribute to this metaflammation chaos [42, 43]. Nonetheless, LPS has already been illustrated relevant to chronic systemic inflammatory reaction. When it comes to NLRP3 inflammasome, LPS acts as the priming signal in the activation process.

## NLRP3 inflammasome and islet β-cells

Activation of the NLRP3 inflammasome is a critical trigger in causing pancreatic islet damage. Prolonged hyperglycemia in pancreatic islets induces ROS accumulation, which results in elevation in TXNIP to activate NLRP3 inflammasome activation and to induce caspase-1-dependent IL-1β maturation [44]. Macrophage infiltration also has been found in the pancreas of high fat diet-induced diabetic rodents, which further promotes IL-1β production and pancreatic cell apoptosis, to compromise islet function and insulin resistance [4, 45]. There is also evidence that elimination of NLRP3 inflammasome-dependent IL-1β production protects islet from fibrosis in obese mice [46]. Moreover, excessive glucose and lipid content can not only cause hyperinsulinemia and insulin resistance, but also result in substantial islet amyloid peptide (IAPP)/amylin expression in infiltrated macrophages [47]. While IAPP/amylin in the β-cells of Langerhans’ islets provides the second signal for NLRP3 inflammasome activation through initiating the interaction between NLRP3 and ASC and subsequent inflammasome assembly [48]. These data demonstrate that NLRP3 inflammasome forms a nexus inflammatory signaling network linking TXNIP, ROS, IAPP and secondary inflammatory cytokines in islet β-cells. Regardless, an important role of of NLRP3 inflammasome in pancreatic islet is strongly supported by a report showing that the insulin secretagogues-glibenclmide inhibits NLRP3 inflammasome activation and decreases proinflammatory cytokine production [49].

## NLRP3 inflammasome and diabetic cardiomyopathy

The pathophysiological mechanism of diabetic cardiomyopathy (DCM), characterized by metabolic disorders, microcirculation disturbance, oxidative stress, cardiomyocyte hypertrophy and apoptosis, myocardial fibrosis, and inflammation, is still not fully clarified. Evidence has shown that silencing of NLRP3 exerts protective effects on DCM in a diabetic rat model [50]. Sustained hyperglycemia and hyperlipidemia can stimulate ROS overproduction, and consequently leading to downstream NLRP3 inflammasome aggregation and activation in cardiomyocytes. In this process, ROS also cause TXNIP dissociation and TRPM2-dependent Ca^2+^ influx which can result in activation of NLRP3 inflammasome. In addition, soluble endogenous ligands like oxidized-low density lipoprotein (ox-LDL) can interact with the pattern-recognition receptor CD36 and elicit lysosome lysis, thus irritates NLRP3 inflammasome activation and IL-1β production [51]. A microbiota metabolite, Trimethylamine-N-oxide (TMAO), recently has been linked with endothelial inflammation and atherosclerosis, due to its ability to initiate lysosomal dysfunction and NLRP3 inflammasome activation [52]. Another approach that mediates inflammasome activation in cardiac fibroblast is the purine type 2 X7 receptors (P2X7R), an ATP-gated non-selective cation channel that opens when stimulated by extracellular ATP, followed by K+ efflux and the formation of pannexin-1. Consequently, various NLRP3 agonists will have access to cytoplasm and promote NLRP3 activation.

Under the circumstance of diabetic metabolic disorder, the aforementioned several mechanisms that induce NLRP3 inflammasome activation can activate pro-caspase-1; The resulting mature caspase-1 will shear pro-IL-1β and pro-IL-18 into their active forms, of which the latter will subsequently set off inflammatory cascade in cardiomyocytes [6]. Those inflammatory factors stimulate myocardial fibroblast to overexpress collagen I and collagen III; collagen accumulation contributes to myocardial fibrosis and cardiac remodeling [53]. In addition, caspase-1-dependent pyroptosis is another NLRP3 inflammasome mediated programmed cell death that promotes the progression of diabetic cardiomyopathy [54].

**Figure 2.**
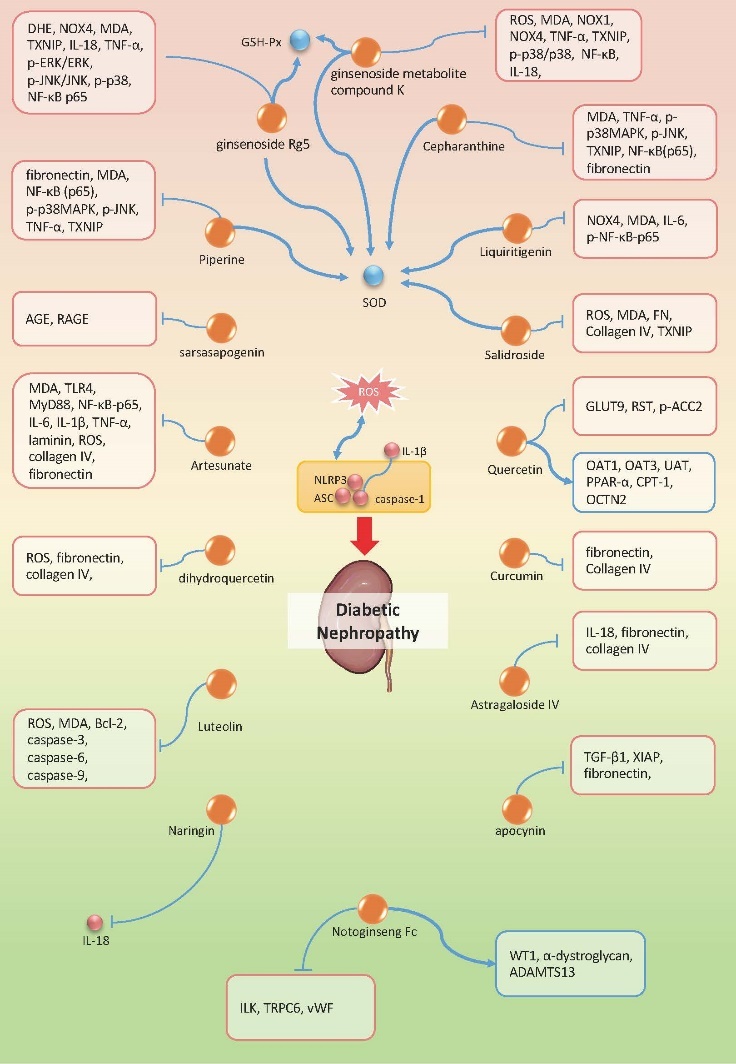
Natural compounds used to treat DN via targeting NLRP3 inflammasome. Orange dots refer to the therapeutic candidates. All the candidates exert inhibition effect on NLRP3 inflammasome. Red dots or molecules shown in red edged text box represent the down-regulated molecules besides NLRP3 inflammasome components by the specific drugs, while the blue ones represent the up-regulated molecules. ↑: promotional effect; ?: inhibitive effect.

## NLRP3 inflammasome and diabetic nephropathy

Diabetic nephropathy (DN) is one of the most common complications among diabetic patients and also the leading cause of end-stage renal failure [55]. The pathological manifestations aggravate with the progression of the disease, including mesangial cell and matrix proliferation, glomerular basement membrane (GBM) thickening, glomerular nodular changes, proximal tubular defects, arteriole hyaline change, and so on [56]. Inflammatory reactions are implicated in the pathogenesis of DN, which damage different types of cells in varying degrees. Enormous evidence has shown that NLRP3 inflammasome-induced microinflammation (also known as “low-grade inflammation”) could directly induce the functional and morphological modifications of podocytes, leading to hypertrophy, detachment, and apoptosis of podocytes, and glomerular sclerosis eventually [57-59]. Inflammasome activation can be detected in glucose-stressed glomerular endothelial cells in vivo and in vitro within glomeruli [60]. Using confocal microscopy, researchers have observed partial colocalization of NLRP3 or cleaved caspase-1 within podocytes and glomerular endothelial cells in diabetic humans or mice, respectively. In addition, other renal cells, such as tubular epithelial cells, can likewise secrete pro-inflammatory cytokines and may thus aggravate diabetic nephropathy [61].

Several inflammatory signaling pathways are closely involved, for example the ROS/TXNIP signaling, nuclear factor-κB (NF-κB) signaling, mitogen-activated protein kinases (MAPK) and nuclear factor erythroid-related factor (Nrf2) signaling, in which NLRP3 acts as the core link and molecular switch in this intricate inflammatory network. Overexpression of TXNIP and NLRP3 can result in higher levels of ROS, MDA and some inflammatory cytokines; whereas TXNIP silencing, or knockdown can inhibit the TXNIP/NLRP3 signaling and ameliorates mesangial cell proliferation and extracellular matrix deposition [62]. Activation of NF-κB signaling upregulates the expression of NLRP3 in response to toll-like receptor (TLR) ligands, which are recognized as the priming signals for inflammasome activation [63]. This is also found in renal tissue, and Nrf2 activation could restrain NF-κB/NLRP3 mediated inflammatory signaling significantly by reducing IκB phosphorylation [64, 65]. As to the MAPK signaling, which includes p38 MAPK, JNK, ERK, researchers have proven that p38 MAPK is responsible for podocytes apoptosis and mesangial proliferation, and NLRP3 inflammasome participates in the p38 MAPK phosphorylation [66].

**Table 1:** *In vivo* study of Chinese medicine treating diabetic complications by inhibition of NLRP3 inflammasome.

Names	Origin Drug Resources	Animal Model	Dosage	Diabetic Complications	Targeted Pathways or pathological mechanisms	Ref
Apocynin	Apocynum venetum L.	SD + STZ	60mg/kg BW	diabetic nephtopathy	NLRP3/XIAP signaling	[77]
Cepharanthine	Senecio scandens Buch. -Ham. ex D.Don	SD + STZ (50mg/kg)	10 mg/kg/day (i.p.)	diabetic nephropathy	MAPK, NF-κB/NLRP3	[67]
Curcumin	Curcuma longa L.	db/db mice	200 mg/kg/day (by gavage)	diabetic nephropathy		[106]
Dihydroquercetin	Pseudotsuga menziesii (Mirb.) Franco	SD + HFD/STZ (30 mg/kg)	25, 50, 100 mg/kg/day	diabetic nephropathy	ROS/TXNIP/NLRP3, NF-κB	[75]
Formononetin	Spatholobus suberectus Dunn	C57BL6/J +STZ (180 mg/kg)	25, 50 mg/kg/bw	diabetic cognitive impairment	HMGB1/TLR4/NF-κB	[96]
Gallic Acid	Phyllanthus emblica L.	Wistar + STZ (50 mg/kg)	25 mg/kg/day (by gavage)		TXNIP/NLRP3 signaling	[107]
Gastrodin	Gastrodia elata Blume	db/db mice	70 and 140 mg/kg (by gavage)	diabetic encephalopathy	ER stress, TXNIP/NLRP3 signaling	[99]
Ginsenoside metabolite compound K	Panax ginseng C.A.Mey.	C57BL6/J + HFD (40 mg/kg)	10, 20, 40 mg/kg/day	diabetic nephropathy	ROS/TXNIP/NLRP3, NF-κB/p38	[74]
Ginsenoside Rg1	Panax ginseng C.A.Mey.	Wistar + STZ (40 mg/kg); C57BL6/J + STZ (130mg/kg)	20 mg/kg/day (i.p.); or 10, 20 and 40 mg/kg (by gavage)	diabetic cardiomyopathy	oxidative stress, mitochondrial biogenesis, AMPK/Nrf2/HO-1; Keap1/Nrf2/HO-1	[81, 93]
Ginsenoside Rg5	Panax ginseng C.A.Mey.	C57BL6/J + HFD/STZ (40 mg/kg)	30, 60mg/kg BW	diabetic nephropathy	MAPK, NF-κB/NLRP3 signaling	[70]
Gypenosides	Gynostemma pentaphyllum (Thunb.) Makino	SD rats + STZ (35 mg/kg)	200 mg/kg (by gavage)	diabetic cardiomyopathy	ROS/NLRP3 signaling	[83]
Isoliquiritigenin	Dianthus chinensis L.	C57BL6/J + HFD	diet supplementation (0.5% w/w)		TLR4/NLRP3 signaling	[108]
Notoginseng Fc	Panax notoginseng (Burkill) F.H.Chen	db/db mice	5 mg/kg/day by gavage	diabetic nephropathy		[78]
Oleanolic acid	Prunella vulgaris L.	SD + STZ (30 mg/kg)	100 mg/kg/d by gavage	diabetic vascular complications		[86]
Palbinone	Paeonia × suffruticosa Andrews	SD + STZ (65 mg/kg)	20 mg/kg/bw	diabetic retinopathy	oxidative stress, Nrf2 pathway	[100]
Piperine	Piper nigrum L.	SD + STZ (51 mg/kg)	30 mg/kg/day (i.p.)	diabetic nephropathy	NF-κB signaling	[67]
Quercetin	Bupleurum chinense DC.	SD + STZ (55,60 mg/kg), or db/db mice	25, 35, 50, 70, 100 mg/kg/day by gavage	diabetic nephropathy; diabetes-associated NAFLD; diabetic encephalopathy; diabetic cardiomyopathy	lipid accumulation; SIRT1/NLRP3	[79, 94, 97, 98, 109]
Salidroside	Rhodiola crenulata (Hook.f. & Thomson) H.Ohba	C57BL6/J +HFD	100 mg/kg/d	diabetes-associated NAFLD	AMPK-dependent TXNIP/NLRP3	[95]
Salvianolic Acid A	Salvia miltiorrhiza Bunge	Zucker diabetic fatty (ZDF) rats + HFD	0.5 or 1 mg/kg b.w., tail vein i.v.	Diabetic atherosclerosis	NF-κB/NLRP3 signaling	[87]
Sarsasapogenin	Anemarrhena asphodeloides Bunge	SD rats + STZ (60 mg/kg)	20, 60 mg/kg BW	diabetic nephropathy	AGEs/RAGE axis	[80]
Sulforaphane	Raphanus raphanistrum subsp. sativus (L.) Domin	SD rats + STZ (65 mg/kg)	0.5, 1 mg/kg/d	diabetic retinopathy	Nrf2 pathway	[101]
Ursolicacid	Eriobotrya japonica (Thunb.) Lindl.	ICR mice + STZ (30 mg/kg)	100 mg/kg (by gavage)	diabetic cardiomyopathy		[53]

## 3. Active compounds from herbal medicine ameliorates diabetes and its complications by targeting NLRP3 inflammasome

Natural derived herbal medicine and their active ingredients have been used for thousands of years in the treatment of diabetes and its complications. The clinical evidence obtained from ethnomedicine physicians has already proven their beneficial effect on symptom relief, postponement of disease progression and safety. However, the underlying mechanisms of their protective role are far from dissolved. Recently, accumulating evidence has shown that various active agents from herbal medicine can ameliorate diabetes and diabetic complications via inhibiting the NLRP3 inflammasome. In order to summarize the literature regarding the active compounds from herbal medicine used to treat diabetes and diabetic complications through inhibiting NLRP3 inflammasome, we searched several databases including PubMed (www.pubmed.com), EMBASE (www.embase.com), Web of Science (www.isiknowledge.com), Chinese National Knowledge Infrastructure (CNKI, www.cnki.net), Chinese Scientific Journal Database (VIP,lib.cqvip.com), Wanfang Database (www.wanfangdata.com.cn) and China Biology Medicine disc (CBMdisc, www.sinomed.ac.cn) with the keywords of "diabetes", "diabetic complications", "NLRP3 inflammasome", “natural compounds” and "herbal medicine" in various combinations. More than 100 scientific papers were consulted by December 2020. A total of 31 active compounds were recognized (Table 1 & 2). All the reports are pre-clinical studies.

### Diabetic Nephropathy

There are 16 ingredients demonstrated to exert protective effect on DN by inhibiting NLRP3 inflammasome (Table 1, Table 2, Fig 2). Cepharanthine, piperine, artesunate, liquiritigenin, ginsenoside Rg5 and ginsenoside metabolite compound K are all claimed to suppress NF-κB-mediated NLRP3 activation [67-71]. Cepharanthine, piperine and ginsenoside Rg5 can also inhibit MAPK signaling. Inhibition of NF-κB (NF-κB p65, p-IKK) and MAPK signaling (p38MAPK, JNK, ERK) usually lead to suppression of downstream inflammatory cytokines, including IL-1β, IL-18, TNF-α, and so on. Next, luteolin, ginsenoside metabolite compound K, artesunate, salidroside and dihydroquercetin played a role in anti-oxidative stress and restrain ROS-mediated NLRP3 activation, accompanied by the downregulation of MDA level [62, 68, 72-75]. And piperine, ginsenoside Rg5, ginsenoside metabolite compound K, cepharanthine, liquiritigenin and salidroside also contribute to restoration of SOD [62, 67, 69, 70, 74, 76]. In addition, apocynin was found to down-regulate the NLRP3/X-linked inhibitor of apoptosis protein (XIAP) signaling and alleviate renal fibrosis in diabetic rats [77]. Q. Yu, et al investigated luteolin’s effect on apoptosis-related proteins including B-cell lymphoma-2 (Bcl-2), caspase-3 and caspase-9, and concluded that luteolin might inhibit NLRP3 inflammasome activated podocyte apoptosis [72]. Notoginseng Fc, a major component and novel saponin isolated from Panax notoginseng (Burkill) F. H. Chen, was shown to exhibit anti-oxidative and anti-inflammatory properties. Researchers found that besides reducing inflammatory reactions, notoginseng Fc also protect from renal fibrosis and podocyte apoptosis by inhibiting ILK and transient receptor potential cation channel 6 (TRPC6) and relieve microcirculation disturbance by modulating a disintegrin-like and metalloprotease with thrombospondin type 1 repeats member 13 (ADAMTS13) and von Willebrand factor (vWF) expression [78]. Dietary flavonoid quercetin has long been recognized as a hypouricemic agent. Chuang W and his co-workers found that quercetin can not only attenuate hyperuricemia through regulation of renal urate transport-related proteins like renal-specific transporter (RST), organic anion transporters 1 (OAT1), glucose transporter 9 (GLUT9), but also be capable of accommodating lipid metabolism-related genes such as peroxisome proliferators activated receptor-α (PPAR-α), carnitine palmitoyltransferase-1 (CPT-1), acetyl-CoA carboxylase-2 (ACC-2) and organic cation transportor 2 (OCTN2), to protect from dyslipidemia [79]. This evidence explained the possible mechanism of quercetin’s anti-inflammasome effect, given that uric acid and saturated fatty acids are both priming signals for NLRP3 inflammasome activation. Further, another research group found that sarsasapogenin from the rhizome of Anemarrhena asphodeloides Bunge can markedly ameliorate early stage DN through inhibition of NLRP3 inflammasome activation and suppression of AGEs-RAGE interaction [80]. In brief, these active agents mainly repress NLRP3 inflammasome components (NLRP3, ASC, caspase-1) expression and its activation through inhibition of NF-κB, MAPK and ROS/TXNIP mediated pathways. Through their anti-inflammatory and anti-oxidative effect, those ingredients decrease the fibronectin, collagen IV and vimentin expression, alleviate renal fibrosis, relieve the inflammatory renal injury and improve renal function.

**Table 2:** *In vivo* study of Chinese medicine treating diabetic complications by inhibition of NLRP3 inflammasome.

Names	Origin Drug Sources	Cell Lines	Dosage	Diabetic Complications	Targeted Pathways or pathological mechanisms	Ref
6-shoqaol	Zingiber officinale Roscoe	Human artery smooth muscle cells (HASMCs) + HG(25mM)	-	diabetic vascular complications	Akt/ROS/NLRP3 inflammasome signaling	[88]
Artesunate	Artemisia annua L.	Rat cell line (HBZY-1) + HG(30mM)	15, 30 μg/ml	diabetic nephropathy	TLR4/NF-κB/NLRP3 inflammasome pathway	[68]
Astragaloside IV	Astragalus mongholicus Bunge	endothelial progenitor cells (EPCs) + ox-LDL(50mM)	10,20,40 μM	diabetic vascular complications	LOX-1/NLRP3 pathway	[92]
Curcumin	Curcuma longa L.	HK-2 cell line + HG(35mM)	5,10,15 μM	diabetic nephropathy		[106]
Dihydroquercetin	Pseudotsuga menziesii (Mirb.) Franco	Rat kidney mesangial cells (HBZY-1)/human proximal renal tubular epithelial cells (HK2) + HG(30mM)	10, 20, 40, 80 μM	diabetic nephropathy	ROS, oxidative stress	[75]
Formononetin	Spatholobus suberectus Dunn	SH-SY5Y cells + HG(33mM)	2.5, 5, 10 μM	diabetic cognitive impairment	HMGB1/TLR4/NF-κB	[96]
Gallic Acid	Phyllanthus emblica L.	INS-1 cells + HG(25mM)	2.5, 5, 10 μM		TXNIP/NLRP3 signaling	[107]
Ginsenoside metabolite compound K	Panax ginseng C.A.Mey.	Rat glomerular mesangial cell line HBZY-1 cells + HG(30mM)	10, 20, 40, 50 μM	diabetic nephropathy	NF-κB/p38	[74]
Gypenosides	Gynostemma pentaphyllum (Thunb.) Makino	H9C2 cells / neonatal rat ventricular myocytes (NRVMs) + HG(25/35mM)	100, 200, 400 mg/L	diabetic cardiomyopathy	ROS/NLRP3 signaling	[83]
Liquiritigenin	Glycyrrhiza uralensis Fisch. ex DC.	rat glomerular mesangial cells (HBZY-1) + HG(30mM)	20, 40 μM	diabetic nephropathy	NF-κB/NLRP3 signaling	[69]
Luteolin	Lonicera japonica Thunb.	Mouse podocyte cell-5 (MPC-5) + HG(30mM)	25, 50, 100 μM	diabetic nephropathy	ROS/NLRP3	[72]
Mangiferin	Anemarrhena asphodeloides Bunge	Human umbilical vein cell line (EA. hy926) + HG(25mM) / TG(1mM)	0.1, 1, 10 μM	diabetic vascular complications	ER stress, TXNIP/NLRP3	[89]
Naringin	Citrus × aurantium L.	Rat glomerular mesangial cells + HG (15, 25, 30, 35, 50 mM glucose)/mannitol (24.4 mM)	5, 10, 20, 40, 80 μM	diabetic nephropathy		[110]
Puerarin	Pueraria montana var. lobata (Willd.) Maesen & S.M.Almeida ex Sanjappa & Predeep	mouse vascular endothelial cell (mMVEC line) + HG(30mM)	1, 10, 25, 50 μM	diabetic vascular complications	ROS/TXNIP/Nlrp3 pathway	[73]
Quercetin	Bupleurum chinense DC.	normal rat hepatocyte line (BRL-3A) / human liver tumour cell line (HepG2) + HG(30mM)	10, 20μM	diabetes-associated NAFLD	TXNIP/NLRP3 signaling	[94]
Rutin	Styphnolobium japonicum (L.) Schott	HUVECs + HG(25mM)	10, 30, 100μM	diabetic vascular complications	ROS/NLRP3 signaling	[90]
Salidroside	Rhodiola crenulata (Hook.f. & Thomson) H.Ohba	Rat glomerular mesangial cell line HBZY-1 + HG(30mM); human umbilical vein endothelial cells (HUVECs) + AGEs (200 μg/ml); Primary hepatocytes from C57BL6/J + (30 mM glucose) +10 nM insulin	20, 40 μM; 10, 50, 100μM; 0.1, 1, 10μM	diabetic nephropathy; diabetic vascular complications; diabetes-associated NAFLD	TXNIP-NLRP3; AMPK/NF-κB/NLRP3; AMPK-dependent TXNIP/NLRP3	[62, 91, 95]
Sulforaphane	Raphanus raphanistrum subsp. sativus (L.) Domin	Rat Müller cell + HG(25mM)	2.5 μM	diabetic retinopathy	oxidative stress, Nrf2 signaling	[101]

### Diabetic Cardiomyopathy

**Figure 3.**
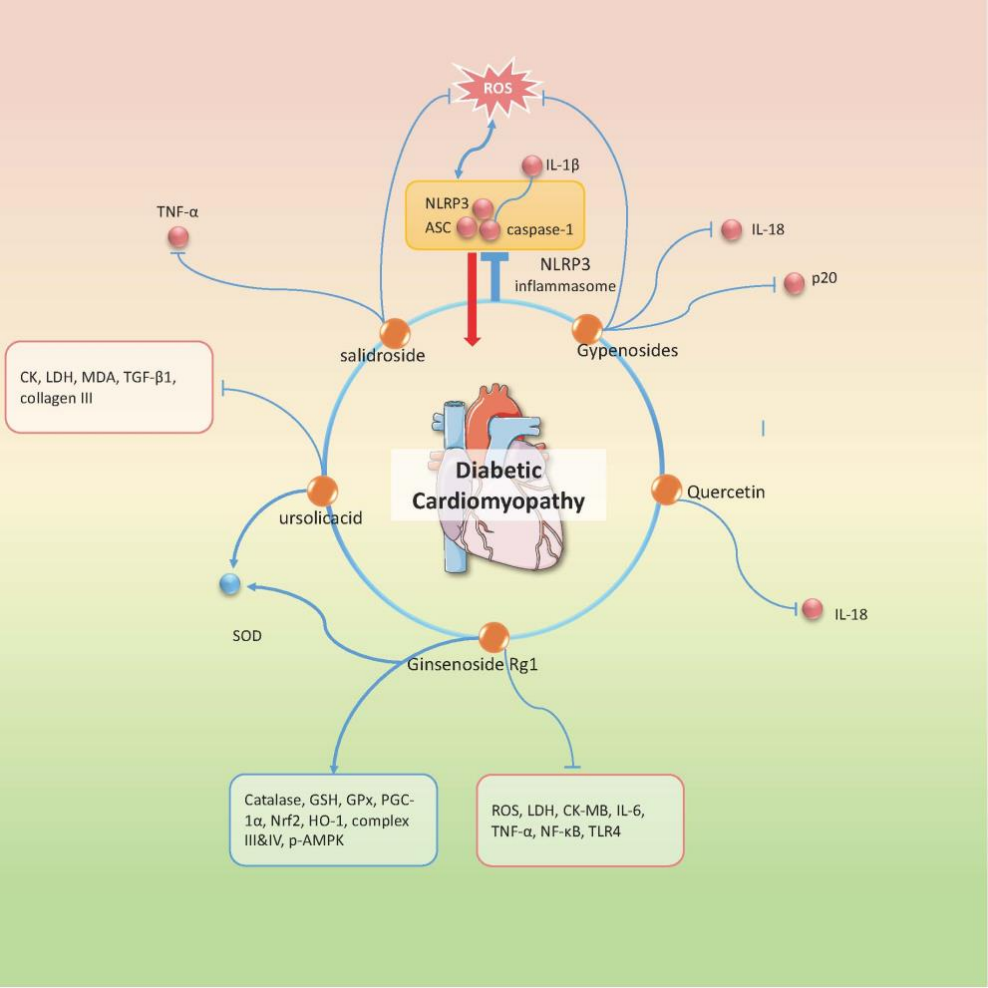
Natural compounds in the treatment of DCM via targeting NLRP3 inflammasome. Orange dots refer to the therapeutic candidates. All the candidates exert inhibition effect on NLRP3 inflammasome. Red dots or molecules shown in red edged text box represent the down-regulated molecules besides NLRP3 inflammasome components by the specific drug, while the blue ones represent the up-regulated molecules. ↑: promotional effect; ⊥: inhibitive effect.

DCM is another diabetic complication whose pathogenesis is closely related to NLRP3-mediated inflammatory reaction. So far there are 5 active agents, ginsenoside Rg1, gypenosides, quercetin, ursolicacid, salidroside confirmed to ameliorate DCM through inhibition of NLRP3 inflammasome (Fig 3). Ginsenoside Rg1 is an active ingredient derived from Panax noto ginseng and known for its cardioprotective effect. A group of researchers conducted an experiment on STZ-induced diabetic rats and claimed that ginsenoside Rg1 treatment decreased the myocardial injury markers such as LDH, CK-MB and AST, suppressed oxidative stress via TLR4/NF-κB signaling and reduced the NLRP3 inflammasome component expressions. The underlying mechanism may be associated with regulation of AMPK/Nrf2/HO-1 signaling pathway, a metabolic stress sensor that exerts beneficial effect on cardiomyocyte and mitochondrial biosynthesis [81]. Gypenosides is one of the major ingredients of Gynostemma pentaphylla (Thunb.) Makino that has anti-hyperglycemia, anti-inflammatory, and cardioprotective properties [82]. A research group utilized HFD/STZ diabetic rats and high glucose stimulated H9C2 cell line to study its effect on NLRP3 inflammasome. Their experiments revealed that high glucose induced excessive production of ROS and cytochrome c influx, leading to NLRP3 activation and secondary inflammatory cytokines release; while gypenosides could reverse this process and attenuate myocardial injury [83]. In addition, salidroside and ursolicacid have been also proven to exert anti-oxidative effect and to inhibit ROS-mediated NLRP3 inflammasome activation in TNF-α induced CMECs, primary cardiomyocytes and STZ induced diabetic mice, respectively [53, 84]. In summary, these natural medicines exhibit remarkable inhibitory effect on induced myocardial fibrosis and inflammation, targeting on NLRP3 in diabetic myocardial tissue mainly through repressing NF-κB or ROS mediated signaling. Moreover, caspase-1 induced pyroptosis is another important pathogenic element in DCM and hitherto there are few studies exploring the effect of natural compounds or herbal medicine on inflammasome related cardiomyocyte programed death in DCM. This sheds light upon the future pharmacological research direction and provides a novel target for treating DCM.

### Diabetic Macrovascular Complications

**Figure 4.**
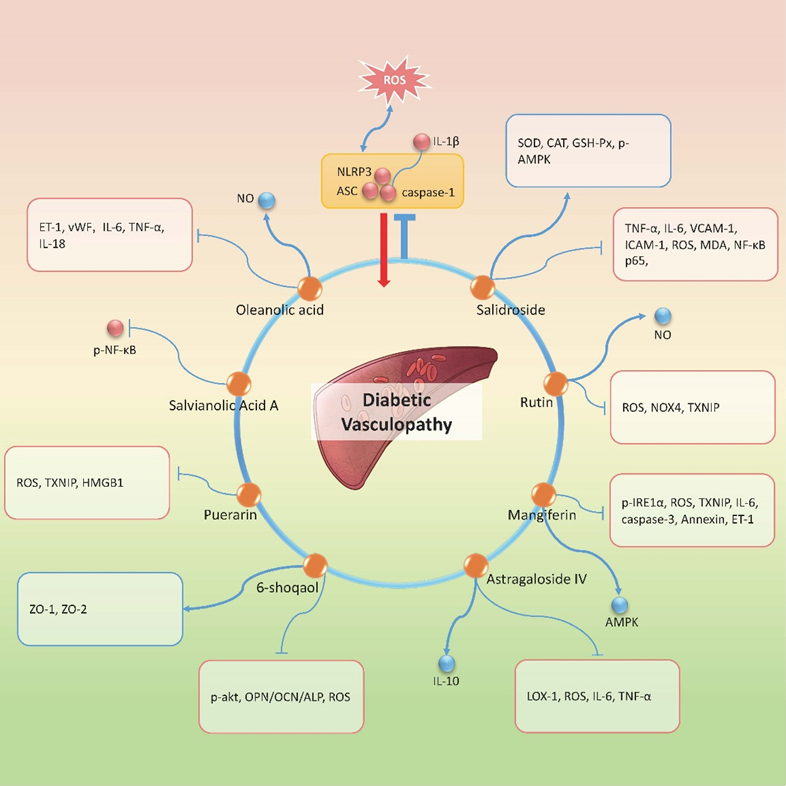
Natural compounds in the treatment of DV via targeting NLRP3 inflammasome. Orange dots refer to the therapeutic candidates. All the candidates exert inhibition effect on NLRP3 inflammasome. Red dots or molecules shown in red edged text box represent the down-regulated molecules besides NLRP3 inflammasome components by the specific drug, while the blue ones represent the up-regulated molecules. ↑: promotional effect; ⊥: inhibitive effect.

Macrovascular complications refer to atherosclerosis of aorta, coronary artery, cerebral basilar artery, peripheral artery and so on. This is among the most common diabetic complications as well as the leading cause of death in diabetic patients because diabetic patients are at higher risk of getting coronary heart disease and cerebrovascular disease [85]. Unlike diabetic microangiopathy, the macrovascular complication is characterized by endothelial dysfunction, atherosclerosis, microvascular basement membrane thickening and glycogen deposition. Vascular endothelium inflammation plays an important role in diabetic vasculopathy. Previous studies have confirmed that hyperglycemia induced NLRP3 inflammasome activation, while the active caspase-1 induces excessive release of inflammatory cytokines, leading to tight junction disruption and consequent endothelial permeability. So far, there are 8 active agents claimed to reduce inflammatory response targeting NLRP3 inflammasome related signaling (Fig 4). Oleanolic acid is a triterpenoid compound from various herbs with multiple biological effects. A study demonstrated that NLRP3 inflammasome played an important role in neointimal hyperplasia and endothelial dysfunction, while oleanolic acid administration attenuated carotid artery injury in diabetic rats through suppressing NLRP3 inflammasome [86]. Besides, Salvianolic acid A also inhibits NF-κB mediated NLRP3 activation in aortic tissues in ZDF rats, and thereby alleviating atherosclerosis at early stage [87]. However, these two studies are not enough to explain the beneficial effects of these compounds, and more detailed data are required to explain the inflammasome-inhibiting effect of the two agents. Antioxidant effect is a generally accepted advantage of herbal medicine. Several studies have shown that some active ingredients in herbs can ameliorate diabetic vascular injury and play anti-inflammatory effect via inhibiting NLRP3 inflammasome signaling. It has been reported that 6-shogal (a major ginger derivate) exhibits beneficial effect on human artery smooth muscle cells (HASMCs). Because 6-shogal treatment inhibits Akt activation, ROS production and consequent NLRP3 inflammasome activation, thus attenuating high-glucose-induced HASMCs calcification [88]. In addition, mangiferin, puerarin, and rutin all can alleviate oxidative stress and reduce inflammation in vascular endothelial cells through blocking ROS/TXNIP/NLRP3 signaling [73, 89, 90]. Salidroside has long been known by its antioxidant effects in a variety of AGEs (advanced glycation end products) -related diseases, and it is also found to ameliorate AGEs-induced endothelial inflammation via regulating the AMPK/NF-κB/NLRP3 signaling [91]. Furthermore, Astragaloside IV from Astragalus mongholicus Bunge and Ginsenoside Rg1 from Panax ginseng C.A.Mey. are also recognized as anti-oxidative agents and potential NLRP3 inflammasome inhibitors [92, 93]. A study showed that ox-LDL could induce lectin-like oxidized LDL receptor 1 (LOX-1) overproduction in endothelial progenitor cells, and the latter triggers ROS-dependent NLRP3 activation. One study has shown that Astragaloside IV treatment could protect endothelial progenitor cell from impairment via downregulation of the LOX-1/NLRP3 pathway [92]; the other study reported that Rg1 resisted against ROS-mediated inflammatory reactions by up-regulating Nrf2/antioxidant response elements (AREs) pathway and improving antioxidant enzyme activities, which all contributed to NLRP3 inflammasome suppression in STZ induced diabetic rats [93]. Taken together, the natural compounds mentioned above mostly have antioxidant properties. Through decreasing ROS production, it can partly explain their role as NLRP3 inflammasome inhibitors. This provides us with novel concept about the complicated relationship between inflammation and oxidation, as well as possible strategies for the treatment of diabetic vascular complications. Moreover, other natural antioxidant molecules could also be tested the antagonism towards NLRP3 inflammasome, which will enrich our understanding of the mechanism underlying the anti-inflammatory effect of herbal medicine.

### Other Diabetic Complications

For other diabetic complications such as diabetic cognitive impairment, diabetic retinopathy, diabetes-associated fatty liver and so on, targeting NLRP3 inflammasome signaling is also proven to be an effective therapeutic approach. Quercetin and salidroside play an active role in treating several diabetic complications. Studies have shown that they also help improve diabetes-associated non-alcohol fatty liver disease (NAFLD) syndrome. In in-vivo experiments in SD rats and in vitro experiments with normal rat hepatocyte line (BRL-3A) and human liver tumor cell line (HepG2), quercetin and allopurinol, by inhibiting TXNIP, can reduce NLRP3 inflammasome activation and subsequently inhibit lipid accumulation [94]. While salidroside was demonstrated to attenuate high-fat diet induced NAFLD via modulation of AMPK-Dependent TXNIP/NLRP3 pathway [95]. To be more specific, salidroside caused abatement in lipid accumulation in liver and hepatocytes, decrease of TXNIP/NLRP3 overexpression, reduction of ACC and AMPK phosphorylation, and restoration of SOD and MDA levels. These findings suggest that salidroside exerts anti-oxidative and anti-inflammatory effects, which makes it suitable for treating not only NAFLD but also other metaflammation-related metabolic disturbance.[Fig F5-ad-12-7-1587]

**Figure 5. F5-ad-12-7-1587:**
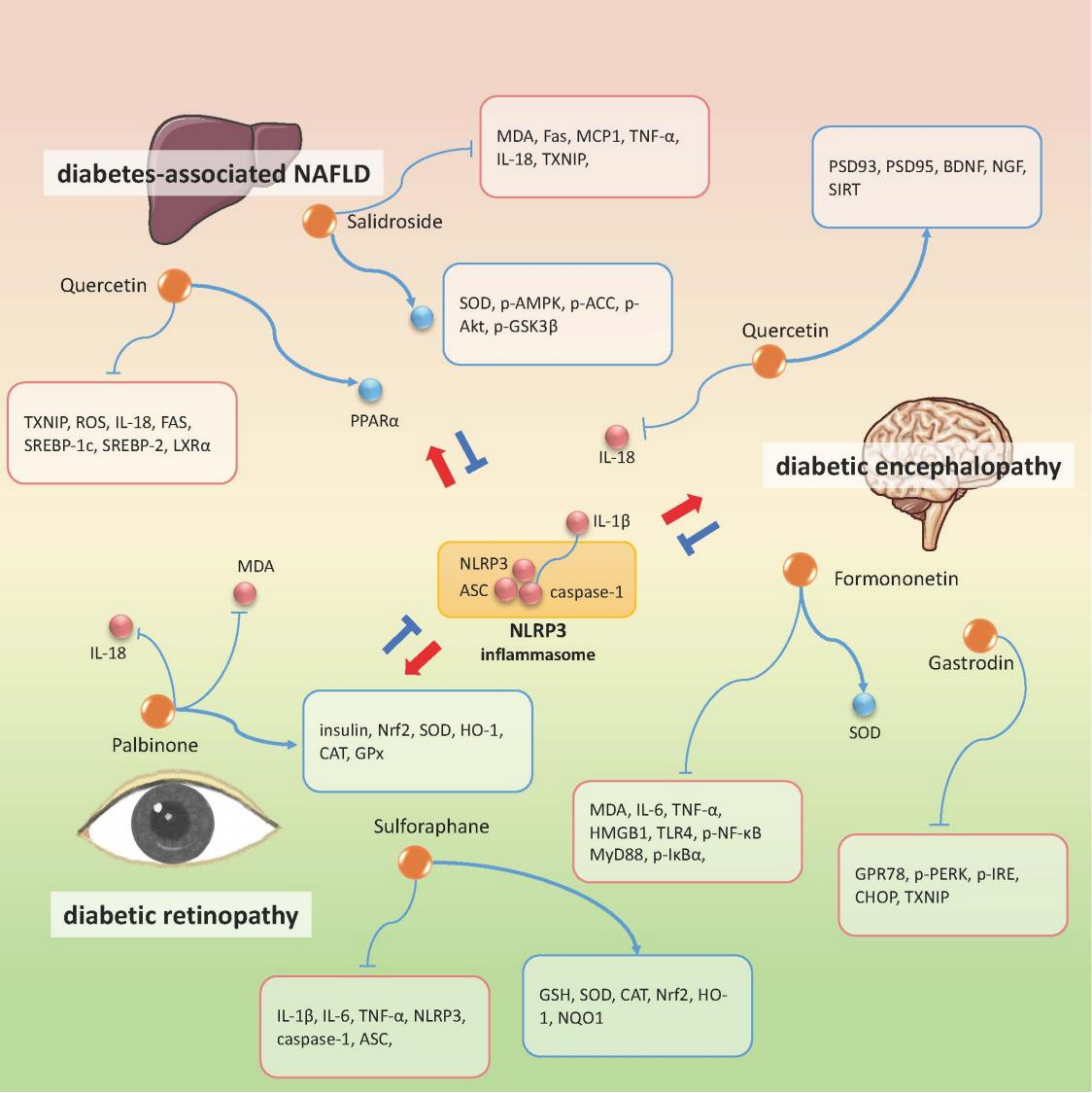
Natural compounds in the treatment of diabetic retinopathy, diabetic encephalopathy, diabetes-associated NAFLD via targeting NLRP3 inflammasome. Orange dots refer to the therapeutic candidates. All the candidates exert inhibition effect on NLRP3 inflammasome. Red dots or molecules shown in red edged text box represent the down-regulated molecules besides NLRP3 inflammasome components by the specific drug, while the blue ones represent the up-regulated molecules. ↑: promotional effect; ⊥: inhibitive effect.

Quercetin, together with formononetin and gastrodin, have been also shown to exert beneficial effects on diabetic encephalopathy (DE) [96-99]. Gastrodin is one of the natural components derived from the Gastrodia elata Blume, a traditional herbal medicine that has long been used in treating hypertension, dementia, and stroke. In db/db mice, gastrodin treatment can improve learning and memory ability and depressive-like behaviors, which was considered through inhibiting ER stress and NLRP3 inflammasome activation in the hippocampus [99]. Formononetin, the main bioactive isoflavones from Spatholobus suberectus Dunn, also targets inflammatory reactions to suppress DE progression, but via high mobility group box-1 protein (HMGB1)/TLR4/NF-κB mediated NLRP3 inflammasome activation [96]. HMGB1 is a well-known neuroinflammation mediator that amplifies inflammatory response by binding to toll-like recepter-4 (TLR-4) and eventually phosphorylates NF-κB to trigger NLRP3 inflammasome assembly. Slightly different from formononetin, quercetin can prevent DE through Sirtuin1 (SIRT1)/NLRP3 pathway [97]. Quercetin increases the expression of nerve/synapse-related proteins such as postsynaptic density 93 (PSD93), PSD95, nerve growth factor (NGF), brain-derived neurotrophic factor (BDNF).. Meanwhile, quercetin also promotes SIRT1 expression which negatively regulates NLRP3 inflammasome, and thus reducing the release of downstream inflammatory cytokines. Other studies have mentioned that palbinone and sulforaphane can attenuate diabetic retinopathy (DR). These two bioactive ingredients both have anti-inflammatory and anti-oxidative properties; the molecular mechanisms of their actions are found to be related to the synergistic effect on upregulating Nrf2 pathway and suppressing NLRP3 inflammasome assembly in retinal tissues, especially in möller cells [100, 101]. Furthermore, one empirical prescription named Jinmaitong has been shown to ameliorate diabetic peripheral neuropathy [102]. In vivo, Jinmaitong can protect SD rats from mechanical allodynia and myelin sheath injury via inhibiting TXNIP/NLRP3 pathway in the peripheral nerve tissues. However, the key effective constituents from the prescription requires further exploration.

Above all, through sorting and summarizing the literatures, active ingredients derived from herbal medicine are identified with anti-NLRP3 inflammasome property. Some of the compounds exert beneficial effects in multiple tissues, all by suppressing NLRP3 inflammasome activation or assembly, and thus could be used in treating various diabetic complications. This inspires us that the low grade metaflammation actually emphasizes the concept of holism, any agents with anti-inflammatory power might be capable of inhibiting inflammasome activity, attenuating the symptoms, and protecting from disease progression. Besides, inflammation and oxidative stress show exceedingly close relationship in many pathophysiological situations [103-105]. There are abundant proteins and cytokines linked with each other forming a sophisticated network; this implies that if we look for an efficient NLRP3 inflammasome inhibitor, more experimental proof from gene editing animals or cell lines are required. Right now, the published data suggest that many candidates from traditional herbal medicine actually exert anti-inflammatory effects through inhibiting upstream signals of NLRP3 including TXNIP and NF-κB; or by fighting against oxidative stress, such as promoting Nfr2 signaling. Inhibition of NLRP3 inflammasome is a likely consequence of this process, although this claim still awaits more studies to be established. Nonetheless, there is still much work to do in order to screen the effective natural agents for combating diabetes. However, it is certain that natural compounds from herbal medicine consist of a very promising candidate library worthy of further investigation for developing effective treatments for diabetes and its associated complications.

## Conclusion

NLRP3 inflammasome forms a nexus binding oxidative stress, ER stress, inflammatory pathways, and pro-inflammatory cytokines. In this review, we analyzed a total of 31 active compounds that exert therapeutic effects on diabetic complications via inhibition of NLRP3 inflammasome. Identification of promising ingredients from traditional herbal medicine targeting NLRP3 inflammasome, on one hand, enriches our understanding about the mechanisms of herbal medicine and their active compounds; on the other hand, inspires us to search the relationship between inflammation and metabolic disorders in different tissues or organs under chronic hyperglycemia, which sheds light on finding novel therapeutic targets as well as potential constituents or therapies for pharmaceutical researches in the treatment of diabetes and diabetic complications.
